# GeneGenie: optimized oligomer design for directed evolution

**DOI:** 10.1093/nar/gku336

**Published:** 2014-04-29

**Authors:** Neil Swainston, Andrew Currin, Philip J. Day, Douglas B. Kell

**Affiliations:** 1 Manchester Institute of Biotechnology, The University of Manchester, Manchester M1 7DN, UK; 2 School of Computer Science, The University of Manchester, Manchester M13 9PL, UK; 3 School of Chemistry, The University of Manchester, Manchester M13 9PL, UK; 4 Faculty of Medical and Human Sciences, The University of Manchester, Manchester M13 9PT, UK

## Abstract

GeneGenie, a new online tool available at http://www.gene-genie.org, is introduced to support the design and self-assembly of synthetic genes and constructs. GeneGenie allows for the design of oligonucleotide cohorts encoding the gene sequence optimized for expression in any suitable host through an intuitive, easy-to-use web interface. The tool ensures consistent oligomer overlapping melting temperatures, minimizes the likelihood of misannealing, optimizes codon usage for expression in a selected host, allows for specification of forward and reverse cloning sequences (for downstream ligation) and also provides support for mutagenesis or directed evolution studies. Directed evolution studies are enabled through the construction of variant libraries via the optional specification of ‘variant codons’, containing mixtures of bases, at any position. For example, specifying the variant codon TNT (where N is any nucleotide) will generate an equimolar mixture of the codons TAT, TCT, TGT and TTT at that position, encoding a mixture of the amino acids Tyr, Ser, Cys and Phe. This facility is demonstrated through the use of GeneGenie to develop and synthesize a library of enhanced green fluorescent protein variants.

## INTRODUCTION

The *de novo* synthesis of genes is becoming increasingly established in synthetic biology and biotechnology as a means of controlling the specific assembly of amino acids producing active proteins. Current approaches involve the synthesis (or purchase) of a number of short oligonucleotides (typically ∼60 bases in length), which can be assembled to form genes and expressed in a host system of interest.

Recent review papers (1,2) discuss existing software for gene optimization, including Gene Designer (3), GeneDesign (4) and DNAWorks (5). Each of these tools has their advantages: Gene Designer, for example, provides a comprehensive application for designing larger synthetic systems whilst GeneDesign has recently been updated to allow for the construction of entire chromosomes (6). However, none of these packages supports the generation of variant libraries to enable directed evolution studies.

Consequently, GeneGenie, a new online tool available at http://www.gene-genie.org, is introduced to support the design of variant libraries of synthetic genes and constructs. GeneGenie shares many features of existing optimization software, allowing for the design of oligonucleotides encoding the gene sequence responsible for the desired protein sequence and optimized for expression in any suitable host through an intuitive, easy-to-use web interface. The tool ensures consistent oligomer overlap melting temperatures, minimizes the likelihood of misannealing and optimizes codon usage for expression in a selected host.

Output oligomers can be assembled using polymerase chain reaction (PCR)-based methods (7) and are fully compatible with our own optimized gene synthesis protocol developed alongside GeneGenie (A. Currin *et al.*, manuscript in preparation). These methods provide highly efficient assembly, permitting expression and functional analysis of genes up to 2 kb in length before sequence verification. This represents a significant improvement over currently established direct gene synthesis methods. Using this integrated wet- and dry-lab approach, the successful synthesis and direct assay of enhanced green fluorescent protein (EGFP) (8) is demonstrated.

Novelties of GeneGenie include the specification of forward and reverse cloning sequences, facilitating the ligation of the designed gene into a vector and its subsequent expression, and the optional specification of ‘variant codons’ at given positions. These variant codons can include both ‘pure’ (A, C, G and T) and mixed bases. Specification of codons including mixed bases allows for variant sequences to be constructed, supporting mutagenesis studies through the generation of variant libraries. For example, specifying the variant codon TNT (where N is any nucleotide) will generate an equimolar mixture of the codons TAT, TCT, TGT and TTT at that position, encoding a mixture of the amino acids Tyr, Ser, Cys and Phe.

The web server is driven by a simple web interface and runs an efficient simulated annealing algorithm to optimize oligomer design from any supplied protein sequence. The web interface links to both the UniProt protein sequence database (9) and the Codon Usage Database (10), is fully documented with help files and requires no user setup.

## MATERIALS AND METHODS

### System architecture

GeneGenie is a two-tiered web application, developed with the Google Web Toolkit (GWT) and written in Java 7, CSS and HTML. The web interface is accessible through a web browser that supports GWT (Firefox, Internet Explorer 6 and above, Safari 5 and above, Chromium and Google Chrome and Opera latest version) and provides the facility for submitting jobs and viewing results. The web server provides an implementation of a novel simulated annealing algorithm for optimizing gene design. Source code is freely available at http://svn.code.sf.net/p/mcisb/code/mcisb-mercedes/.

### Algorithm description

A novel simulated annealing algorithm (11,12) was developed to optimize gene design. This is described in depth below.

#### Initialization

The job is initialized through the following steps.

- Back translation of the ‘protein sequence’, using codons selected randomly following a Monte Carlo approach according to their frequency in the codon usage table for the selected host organism. The sequence is checked to ensure that it adheres to the specified ‘maximum number of repeating nucleotides’. If the sequence contains more than the specified ‘maximum number of repeating nucleotides’, this process is repeated up to 1000 times until an acceptable initial deoxyribonucleic acid (DNA) sequence is generated.
- If ‘variant codons’ have been selected, these are substituted into the initial DNA sequence, and the replaced codon, encoding the original amino acid at that position, is retained.
- Oligomers are generated, each with a length of the supplied ‘maximum oligo length’ minus a fixed value (currently 5 bp), which provides scope for oligomer lengths to subsequently both be increased and decreased during the optimization process. Overlapping regions are generated by specifying start and end positions such that each overlap has a melting temperature close to the supplied ‘melting temperature’; see ‘melting temperature calculation’, below.
- Upon definition of overlapping regions, the viability of ‘variant codons’ is checked. If a ‘fixed codon’ falls within an overlapping region, it is replaced by the codon that encoded the original amino acid in that position in step (i).
- The initial solution is scored, according to ‘Scoring’, below, and the initial score for each objective [initial Codon Adaptation Index (CAI) score (${\rm CAI}_{{\rm init}}$), initial overlap melting temperature score (${\rm Tm}_{{\rm init}}$), initial misanneal score (${\rm mis}_{{\rm init}}$) and initial fixed codon viability score (${\rm fixed}_{{\rm init}}$)] is retained.

#### Scoring

A solution is scored according to the following criteria. Three objectives are scored for each job—CAI, overlap melting temperatures and misanneals—and a fourth, fixed codon viability is considered if ‘variant codons’ have been selected.

CAI score, ${\rm CAI}_{\rm s}$, is simply defined as $1 - {\rm CAI}$. (See below for the definition of ${\rm CAI}$). Overlap melting temperature score, ${\rm Tm}_{\rm s}$, is calculated as the coefficient of variation of the overlap melting temperatures, ${\rm Tm}_i$, from the target melting temperature, ${\rm Tm}_{\rm t}$. Melting temperatures are calculated as described below:

\begin{equation*} {\rm Tm}_{\rm s} = \frac{{\sqrt {\frac{1}{n}\mathop \sum \nolimits_{i = 1}^n ({\rm Tm}_i - {\rm Tm}_{\rm t} )^2 } }}{{{\rm Tm}_{\rm t} }}.\end{equation*}

The misanneals score, ${\rm mis}_{\rm s} ,$ is calculated as $1 - Z_{{\rm score}}$ of the melting temperatures of the set of positive annealing sequences (that is, those of the oligo overlaps) and the melting temperatures of the set of negative, misannealing sequences:

\begin{equation*} {\rm mis}_{\rm s} = \frac{{3(\sigma _p + \sigma _n )}}{{\left| {\mu _p - \mu _n } \right|}}.\end{equation*}

The positive set is simply the calculated melted temperatures of the overlapping sequences, as described previously. The negative set is generated by calculating melting temperatures between all segments of the gene sequence in both the forward and reverse directions and retaining those within 25°C of the target melting temperature, ${\rm Tm}_{\rm t}$.

The fixed codon viability score, ${\rm fixed}_{\rm s}$, is simply a count of the number of unviable variant codons, that is, requested variant codons that fall in overlapping regions of the sequence.

The overall score, ${\rm score}_{\rm s}$, is the mean of the score of each objective scaled by its corresponding initial score:

\begin{eqnarray*} &&{{\rm score}_{\rm s} =} \\ &&\frac{1}{4} \mathop \sum \left( \frac{{\rm CAI}_{\rm s}}{{\rm CAI}_{{\rm init}}} + \frac{{\rm Tm}_{\rm s}}{{\rm Tm}_{{\rm init}}} + \frac{{\rm mis}_{\rm s}}{{\rm mis}_{{\rm init}}} + \frac{{\rm fixed}_{\rm s}}{{\rm fixed}_{{\rm init}}} \right). \\ \end{eqnarray*}

As a consequence, ${\rm score}_{\rm s}$ of the initial solution is 1, and scores of subsequent solutions are therefore a measure of optimality relative to the initial solution. The simulated annealing algorithm attempts to minimize ${\rm score}_{\rm s}$.

#### Melting temperature calculation

The melting temperature (Tm) calculation utilizes the programming library MELTING (13), applying the following formula of Wetmur (14):

\begin{eqnarray*} &&{\rm Tm} = 81.5 + 16.6{\rm log}_{10} \left( {\frac{{[{\rm Na}_{{\rm equiv}} ]}}{{1 + 0.7[{\rm Na}_{{\rm equiv}} ]}}} \right) + \\ &&0.41 \times \% {\rm GC} - \frac{{500}}{L} - \% {\rm MM},\end{eqnarray*}

where $[{\rm Na}_{{\rm equiv}} ]$ is the equivalent sodium ion concentration and is given below; %GC is the percentage GC content of the sequence; *L* is the sequence length; and %MM is the percentage mismatch.

The equivalent sodium ion concentration is given by von Ahsen *et al.* (15), according to the formula

\begin{eqnarray*} &&[{\rm Na}_{{\rm equiv}} ] = \\ &&[{\rm Na}^ + ] + [{\rm K}^ + ] + \frac{{[{\rm Tris}]}}{2} + 3.79\sqrt {[{\rm Mg}^{2 + } ] - [{\rm dNTP}{\rm }]}. \end{eqnarray*}

#### CAI calculation

The CAI (16) provides a measure of the deviation of the set of codons of the protein encoding region of the optimized gene with respect to a reference set of codons from genes that are highly expressed in the selected host organism. CAI is the geometric mean of the relative adaptiveness, *w_i_*, of each codon, where *w_i_* is the ratio of the codon frequency over that of the most frequently used synonymous codon for a given amino acid:

\begin{equation*} {\rm CAI} = {\rm exp}\left( {\frac{1}{L}\mathop \sum \limits_{l = 1}^L {\rm ln}(w_i (l))} \right)\end{equation*}

\begin{equation*} w_i = \frac{{f_i }}{{{\rm max}{\rm }(f_j )}}\quad i,j \in [{\rm synonymous\;codons\;for\;aminoacid}].\end{equation*}

A CAI of 1 would indicate a DNA sequence in which all codons were the most frequently observed in highly expressed genes for a given amino acid, with the inference that such a sequence would also be highly expressed due to optimal codon usage. Note that, in GeneGenie, calculation of CAI ignores codons that contain degenerate bases, which may arise due to specification of variant codons.

#### Iterations and annealing schedule

The algorithm makes a user-specified number of iterations, following an annealing schedule in which the annealing temperature, *T*, is geometrically cooled to zero from a value at which ∼10% of higher scoring (worse) neighbour solution is accepted. Upon each iteration, the current solution (with score ${\rm score}_{{\rm current}}$) is mutated to form a neighbour solution, which is scored as described above (${\rm score}_{{\rm neighbour}}$) and accepted according to an acceptance probability function $P({\rm score}_{{\rm current}} ,{\rm score}_{{\rm neighbour}} ,T)$ of 1 if score_neighbour_ < score_current_, and ${\rm exp}( - ({\rm score}_{{\rm neighbour}} - {\rm score}_{{\rm current}} )/T)$ otherwise.

#### Mutation

Mutations to the solution take two forms: mutation to the selected codons and mutations to the oligomer start and end positions. At each iteration, oligomer start and end positions are mutated with a probability given by the supplied ‘oligo length mutation rate’ parameter. Start and end positions are mutated by ±10 bp, taken randomly from a uniform distribution. The effect of a mutation on an oligo start or end position is the redistribution of overlapping regions. The solution is therefore rechecked to determine whether requested variant codons now fall within overlapping sequences. If so, they are replaced with a codon encoding the original amino acid at that position. Alternatively, if a fixed codon site now falls within a non-overlapping sequence, the requested fixed codon is set in that position.

Non-variant codons are mutated with a probability given by the supplied ‘codon mutation rate’ parameter. If the codon is mutated, a synonymous codon is selected with a probability given by its frequency in the codon usage table of highly expressed genes in the host organism. The mutation will be rejected if the resulting sequence exceeds the specified ‘maximum number of repeating nucleotides’ parameter.

### Design of a library of variants of EGFP

The EGFP amino acid sequence was input to GeneGenie together with 5′ and 3′ DNA cloning sequences (for sequences used, see Supplementary Data). Variant codons were specified as YAT at position 66 and TWT at position 145. The parameters were set as: ‘maximum oligo length’: 60; ‘melting temperature’: 60°C; host organism: *Escherichia coli*. All other parameters were the default. Results are available at http://www.gene-genie.org?jobId=EE28A988-1587-493D-8A39-4C39777F1F28.

GeneGenie generated 20 oligonucleotide sequences (see Supplementary Data), which were synthesized by Integrated DNA Technologies (Coralville, IA, USA). Oligonucleotides were assembled separately in two sections (1–10 and 11–20) using PCR-based methods (17). In short, a 600-nM mixture containing oligonucleotides 2–9 or 12–19 was made and used as the template for PCR. The oligonucleotides 1 and 10 were used as primers for the first block assembly and 11 and 20 for the second assembly. PCR was done using the Q5 Hot Start polymerase reagents (New England Biolabs, Ipswich, MA, USA) with the following conditions: 98°C for 2 min, then 35 cycles of 98°C for 10 s, 60°C for 30 s (0.5°C/second ramp rate). (In this protocol, the 60°C step is sufficient for both annealing and elongation as the amplicon is small.) Removal of erroneous sequences was performed using Surveyor nuclease (Transgenomic Inc., Omaha, NE, USA) for 2 h at 42°C. Digested products were then used as the template for a second PCR reaction (using oligonucleotides 1 and 20 as primers) to assemble the full EGFP sequence. The oligonucleotides containing the variant codons were supplemented into the reaction to facilitate assembly (concentration 6 nM). PCR conditions were: 98°C for 2 min, then 35 cycles of 98°C for 10 s, 60°C for 20 s and 72°C for 40 s. Full-length EGFP sequences were purified by gel extraction following electrophoresis and then ligated into a pET16b expression vector (Novagen, Madison, WI, USA) using the In-Fusion system (Clontech, Mountain View, CA, USA). Plasmids were transformed into T7 expression *E. coli* cells, incubated overnight at 30°C and expression was induced using 1-mM IPTG. For full details on the gene synthesis method used see A. Currin *et al.*, manuscript in preparation.

## RESULTS

GeneGenie queries are submitted through a simple web interface, including formatted examples, which also allows the input of all parameters required for optimization. Many of these parameters are described below, and details of how they are used by the optimization algorithm are given in the Materials and Methods section.

### Inputs

#### Sequence

The sequence to be optimized comprises three components: two optional cloning sequences (at the 5′ and 3′ ends of the gene), used for ligation into expression plasmids or assembly into larger DNA constructs, and the protein amino acid sequence itself. For protein expression applications, the 5′ coding sequence must be specified such that the coding sequence of the gene will be in the correct reading frame after cloning and may contain a codon encoding a starting methionine if required. The 3′ coding sequence will typically include the specification of one or more stop codons immediately after the coding sequence of the gene. These sequences are fixed DNA sequences and will not be optimized (or otherwise varied) by GeneGenie. However, GeneGenie ‘will’ optimize the protein coding sequence such that misannealing events between these fixed cloning sequences and the coding sequence will be minimized. The ‘protein sequence’ to be optimized can be entered manually or retrieved automatically through an integrated search of the UniProt database. Irrespective of whether the sequence was added manually or extracted from UniProt, the sequence itself may still be modified subsequently.

#### Variant codons

A key feature of GeneGenie is its ability to allow desired codons to be specified at any given position in the protein sequence. These codons may include any combination of ‘pure’ and mixed bases (see Supplementary Data). Codons containing mixed bases may consequently encode a mixture of amino acids. As such, these user-specified codons are named ‘variant codons’. Multiple variant codons will therefore encode a mixture of protein sequences, supporting directed evolution studies through the generation of variant libraries. These variant codons will be fixed by GeneGenie and will not be mutated during the optimization process. However, GeneGenie will attempt to optimize the solution such that requested variant codons fall in non-overlapping sequences. (Overlapping sequences must not contain variant base sequences, as their presence will prevent successful assembly). In cases where the solution cannot be optimized such that a variant codon is placed in a non-overlapping sequence, GeneGenie will revert to the original amino acid at that position and will optimize its codon selection as normal.

#### Melting temperature and buffer concentrations

Successful annealing of oligomers is dependent upon consistent melting temperatures of overlapping sequences (7). This target melting temperature must be specified, allowing GeneGenie to optimize the oligomer design such that all overlapping sequences are homogeneous and close to the specified value. The calculation of melting temperatures is dependent upon the concentration of components within the buffer in which assembly will take place. Concentrations of the ions Na^+^, K^+^ and Mg^2+^ along with that of Tris and deoxyribonucleotide triphosphate (dNTP) can be specified.

A further optimization that GeneGenie performs is that of the oligo lengths. Synthesized oligomers typically have a length limit above which the likelihood of incorporating errors increases. Specifying a ‘maximum oligo length’ can mitigate this issue. This value has a default of 60 nt but has no upper limit.

GeneGenie optimizes codon usage to enable successful expression in the target ‘host organism’. This is dependent upon codon usage tables containing codon frequencies of highly expressed genes in the given organism. These codon usage tables are extracted automatically from the Codon Usage Database upon specification of a host organism. This database currently consists of over 35 000 codon usage tables.

Additional advanced parameters may be specified to modify the optimization. These include ‘number of iterations’, ‘codon mutation rate’ and ‘oligo length mutation rate’. Furthermore, a ‘maximum number of repeating nucleotides’ can be supplied to prevent long stretches of repeating nucleotides, the presence of which may prevent successful assembly and also pose problems for subsequent sequencing of variant libraries.

### Progress of a run observed via the GeneGenie dashboard

**Figure 1.**
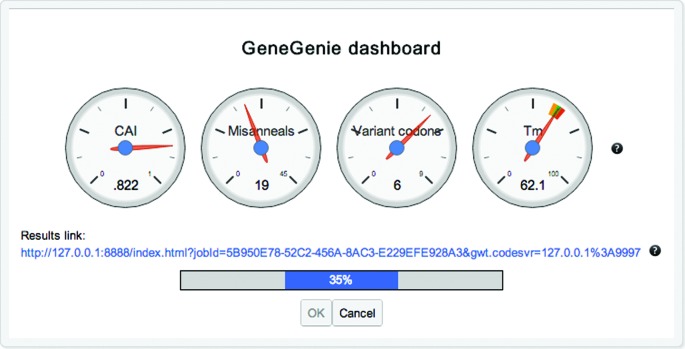
The GeneGenie dashboard, indicating progress of the job, and current score for each of the objectives being optimized.

The GeneGenie dashboard is displayed during the optimization process, indicating the current progress of the job along with the score of each of the objectives that are being optimized. A hyperlink provides a temporary link to the results, allowing these to be accessed subsequently. The optimization job may also be cancelled via this interface (see Figure 1).

### Outputs

Upon completion of a job, results are displayed on a tabular panel in the GeneGenie interface. Multiple results can therefore be viewed and compared. The results are displayed according to the fields described below, along with the original query.

#### Alignment

**Figure 2.**
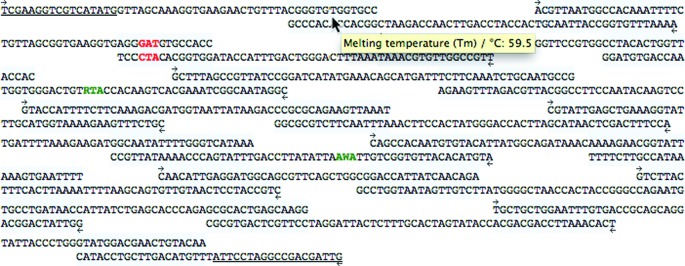
GeneGenie results, displaying the alignment of the optimized set of oligomers.

The alignment of the designed oligomers is displayed. The forward or reverse direction of each oligomer is indicated, and overlapping regions are highlighted. The calculated melting temperature of overlapping sequences can be viewed via a tool tip. If 5′ and 3′ cloning sequences have been submitted, these are indicated with underlines. Requested variant codons are highlighted in red or green font, respectively, depending upon whether they could be specified in the optimized solution. Green codons indicate that the codon was optimized to fall in a non-overlapping sequence, and could therefore be specified. The number of successfully specified variant codons is given in the ‘number of variant codons’ field. Red codons indicate requested variant codons that fall in overlapping sequences. In these cases, the variant codon is rejected and instead, an optimized codon is selected to encode the original amino acid at that position (see Figure 2).

#### CAI

The CAI (16) for the optimized coding sequence is provided (see the Materials and Methods section).

#### Oligos

A table of all oligomers required to construct the gene is given. The table indicates the oligo number, name, direction, sequence and length. The table may be exported in a number of formats, including as CSV, the Synthetic Biology Open Language (SBOL) (18), and in a proprietary format of Integrated DNA Technologies (Coralville, IA, USA), allowing the output of GeneGenie to be submitted directly to a suitable manufacturer for synthesis.

#### Mean melting temperature (Tm, °C) and melting temperatures (Tm, °C)

It displays the mean melting temperature of all overlapping sequences, along with their individual values.

#### Misanneals

Potential misanneal sites, along with repeating sequences that may impede correct assembly, are reported. Potential misanneal sites are defined as being segments of forward and reverse strand sequences that have a melting temperature within 25°C of the target melting temperature for overlapping sequences.

The ‘DNA sequence’, including cloning sequences and the coding sequence, and a ‘Results link’ allowing for the subsequent access of the results, are also provided.

### Design of a library of variants of EGFP

To demonstrate the use of GeneGenie in generating protein variant libraries, a collection of EGFP variants was generated and synthesized. In this demonstration case, the amino acid substitutions that give rise to a ‘blue fluorescent protein’ variant were already known (19)—specifically, the double mutant Y66H/Y145F (20)—and these were targeted in an experiment to optimize a library encoding both the wild type and the blue variant.

Encoding the double mutant involved the specification of two variant codons, encoding both Y and H and Y and F, at positions 66 and 145, respectively. By considering both the standard codon table and variable codons, it can be determined that the codon YAT (i.e. a 1:1 mixture of TAT and CAT) will encode an equimolar mixture of Y and F. Similarly, TWT will encode an equimolar mixture of Y and F. These codons were specified in a GeneGenie optimization for expression of an EGFP variant library in *E. coli*. GeneGenie was able to optimize a set of oligomers within the specified maximum length of 60 bp, with a mean melting temperature of overlapping sequences within 0.3°C of the target melting temperature, a CAI of 0.929 and an absence of potential misannealing sites. Furthermore, the position and lengths of the overlapping sequences were optimized such that both requested variant codons fell within non-overlapping regions. The results of the optimization are available at http://www.gene-genie.org?jobId=EE28A988-1587-493D-8A39-4C39777F1F28.

**Figure 3.**
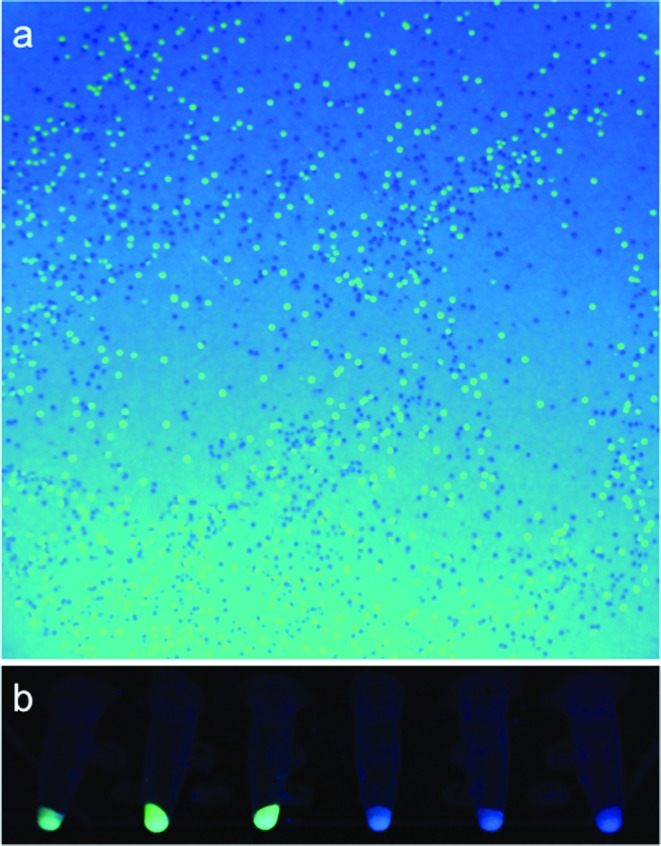
*E. coli* colonies expressing the synthesized EGFP and EBFP variant sequences. (**a**) Under UV light the green and blue colonies can clearly be identified. The image also indicates that the designed gene has been synthesized and expressed with high efficiency, with few negative colonies. Selected colonies from the plate were grown in liquid culture and induced using 1-mM IPTG. Cell pellets are shown in (**b**) under UV light and show the expression of wild-type EGFP (pellets 1–3) and double mutant Y66H/Y145F EBFP (pellets 4–6).

The designed oligomers were purchased, assembled and the resulting genes expressed in *E. coli*. (see the Materials and Methods section). This resulted in a collection of colonies consisting of all four variants (i.e. wild-type EGFP, the single mutants Y66H and Y145F and the double mutant Y66H/Y145F). As expected, when visualized under ultraviolet (UV), the colonies consisted of a mixture of green, wild-type EGFP and blue double mutant ‘blue fluorescent protein’ (EBFP) (see Figure 3). EGFP and EBFP gene sequences were verified as correct using DNA sequencing.

## DISCUSSION

GeneGenie was designed specifically with the support of synthetic biology studied for directed evolution in mind. The example given here, showing the generation of a small library of EGFP variants, provides a simple illustration of the use of carefully chosen but variable nucleotides in particular codons. It is clear from the EGFP example that specifying a codon that encodes *x* amino acids at *y* different sites may generate a library of *x^y^* variants. Specifying the variant codon NCN (which encodes four amino acids) at each of the 10 sites can generate a library of 1 048 576 protein variants. The great advantage of the synthetic biology approach over alternative methods such as sloppy or error-prone PCR is that one can use high effective mutation rates whilst not allowing the premature appearance of stop codons (21,22).

Thus, the use of high-throughput screening and sequencing of variant libraries, along with the targeted mutagenesis that GeneGenie provides, may therefore be used to develop a controlled and iterative approach to directed evolution.

## SUPPLEMENTARY DATA

Supplementary Data are available at NAR Online.

Supplementary Data

## References

[1] Czar M.J., Anderson J.C., Bader J.S., Peccoud J. (2009). Gene synthesis demystified. Trends Biotechnol..

[2] Marchisio M.A., Stelling J. (2009). Computational design tools for synthetic biology. Curr. Opin. Biotechnol..

[3] Villalobos A., Ness J.E., Gustafsson C., Minshull J., Govindarajan S. (2006). Gene Designer: a synthetic biology tool for constructing artificial DNA segments. BMC Bioinformatics.

[4] Richardson S.M., Wheelan S.J., Yarrington R.M., Boeke J,D. (2006). GeneDesign: rapid, automated design of multikilobase synthetic genes. Genome Res..

[5] Hoover D.M., Lubkowski J. (2002). DNAWorks: an automated method for designing oligonucleotides for PCR-based gene synthesis. Nucleic Acids Res..

[6] Richardson S.M., Nunley P.W., Yarrington R.M., Boeke J.D., Bader J.S. (2010). GeneDesign 3.0 is an updated synthetic biology toolkit. Nucleic Acids Res..

[7] Xiong A.S., Yao Q.H., Peng R.H., Li X., Fan H.Q., Cheng Z.M., Li Y. (2004). A simple, rapid, high-fidelity and cost-effective PCR-based two-step DNA synthesis method for long gene sequences. Nucleic Acids Res..

[8] Prasher D.C., Eckenrode V.K., Ward W.W., Prendergast F.G., Cormier M.J. (1992). Primary structure of the Aequorea victoria green-fluorescent protein. Gene.

[9] Apweiler R., Bairoch A., Wu C.H., Barker W.C., Boeckmann B., Ferro S., Gasteiger E., Huang H., Lopez R., Magrane M. (2004). UniProt: the Universal Protein knowledgebase. Nucleic Acids Res..

[10] Nakamura Y., Gojobori T., Ikemura T. (2002). Codon usage tabulated from international DNA sequence databases: status for the year 2000. Nucleic Acids Res..

[11] Kirkpatrick S., Gelatt C.D., Vecchi M.P. (1983). Optimization by simulated annealing. Science.

[12] Černý V. (1985). Thermodynamical approach to the traveling salesman problem: an efficient simulation algorithm. J. Optimization Theory Appl..

[13] Dumousseau M., Rodriguez N., Juty N., Le Novère N. (2012). MELTING, a flexible platform to predict the melting temperatures of nucleic acids. BMC Bioinformatics.

[14] Wetmur J.G. (1991). DNA probes: applications of the principles of nucleic acid hybridization. Crit. Rev. Biochem. Mol. Biol..

[15] von Ahsen N., Wittwer C.T., Schütz E. (2001). Oligonucleotide melting temperatures under PCR conditions: nearest-neighbor corrections for Mg(2+), deoxynucleotide triphosphate, and dimethyl sulfoxide concentrations with comparison to alternative empirical formulas. Clin. Chem..

[16] Sharp P.M., Li W.H. (1987). The codon adaptation index—a measure of directional synonymous codon usage bias, and its potential applications. Nucleic Acids Res..

[17] Xiong A.S., Yao Q.H., Peng R.H., Duan H., Li X., Fan H.Q., Cheng Z.M., Li Y. (2006). PCR-based accurate synthesis of long DNA sequences. Nat. Protoc..

[18] Galdzicki M., Rodriguez C., Chandran D., Sauro H.M., Gennari J.H. (2011). Standard biological parts knowledgebase. PLoS ONE.

[19] Yang T.T., Sinai P., Green G., Kitts P.A., Chen Y.T., Lybarger L., Chervenak R., Patterson G.H., Piston D.W., Kain S.R. (1998). Improved fluorescence and dual color detection with enhanced blue and green variants of the green fluorescent protein. J. Biol. Chem..

[20] Heim R., Tsien R.Y. (1996). Engineering green fluorescent protein for improved brightness, longer wavelengths and fluorescence resonance energy transfer. Curr. Biol..

[21] Moore G.L., Maranas C.D. (2002). eCodonOpt: a systematic computational framework for optimizing codon usage in directed evolution experiments. Nucleic Acids Res..

[22] Pritchard L., Corne D., Kell D., Rowland J., Winson M. (2005). A general model of error-prone PCR. J. Theor. Biol..

