# Supplementary material for: GeneGenie: optimized oligomer design for directed evolution
Source: Nucleic Acids Res. 2014 Apr 29;42(Web Server issue):W395–400. doi: 10.1093/nar/gku336 (PMC4086129; doi:10.1093/nar/gku336)
Supplement: Supplementary Data [file supp_42_W1_W395__index.html]

Supplementary Data 

# GeneGenie: optimized oligomer design for directed evolution

## Supplementary Data

**Files in this Data Supplement:**

- SUPPLEMENTARY DATA
