# Supplementary material for: GeneGenie: optimized oligomer design for directed evolution
Source: Nucleic Acids Res. 2014 Apr 29;42(Web Server issue):W395–400. doi: 10.1093/nar/gku336 (PMC4086129; doi:10.1093/nar/gku336)
Supplement: Supplementary Data [file supp_gku336_nar-00248-web-b-2014-File005.docx]

## Supplementary data 1: Mixed nucleotides

Any combination of 'pure' and mixed nucleotides can be specified as Variant Codons. The use of mixed nucleotides in a Variant Codon allows for the generation of variant libraries, with codons containing mixed nucleotides encoding a mixture of amino acids at that site.

The full list of supported 'pure' and mixed nucleotides is given below:

| **Symbol** | **Description** | **Bases represented** | | | |
| --- | --- | --- | --- | --- | --- |
| **A** | **a**denine | A |  |  |  |
| **C** | **c**ytosine |  | C |  |  |
| **G** | **g**uanine |  |  | G |  |
| **T** | **t**hymine |  |  |  | T |
| **W** | **w**eak | A |  |  | T |
| **S** | **s**trong |  | C | G |  |
| **M** | a**m**ino | A | C |  |  |
| **K** | **k**eto |  |  | G | T |
| **R** | pu**r**ine | A |  | G |  |
| **Y** | p**y**rimidine |  | C |  | T |
| **B** | not A |  | C | G | T |
| **D** | not C | A |  | G | T |
| **H** | not G | A | C |  | T |
| **V** | not T | A | C | G |  |
| **N** | a**n**y base | A | C | G | T |

## Supplementary data 2: EGFP sequences

The EGFP sequence used to demonstrate GeneGenie is given as:

VSKGEELFTG VVPILVELDG DVNGHKFSVS GEGEGDATYG KLTLKFICTT

GKLPVPWPTL VTTLTYGVQC FSRYPDHMKQ HDFFKSAMPE GYVQERTIFF

KDDGNYKTRA EVKFEGDTLV NRIELKGIDF KEDGNILGHK LEYNYNSHNV

YIMADKQKNG IKVNFKIRHN IEDGSVQLAD HYQQNTPIGD GPVLLPDNHY

LSTQSALSKD PNEKRDHMVL LEFVTAAGIT LGMDELYK

The 5’ cloning sequence was specified as:

TCGAAGGTCGTCATATG

The 3’ cloning sequence was specified as follows, including a stop codon:

TAAGGATCCGGCTGCTAAC

## Supplementary data 3: EGFP oligomers

GeneGenie optimised the above EGFP sequence to generate the following cohort of oligomers:

|  | **F/R** | **Sequence** | **Len** |
| --- | --- | --- | --- |
| 1 | F | TCGAAGGTCGTCATATGGTTAGCAAAGGTGAAGAACTGTTTACGGGTGTGGTGCC | 55 |
| 2 | R | AAAATTTGTGGCCATTAACGTCACCATCCAGTTCAACCAGAATCGGCACCACACCCG | 57 |
| 3 | F | ACGTTAATGGCCACAAATTTTCTGTTAGCGGTGAAGGTGAGGGCGATGCCACC | 53 |
| 4 | R | TTGCCGGTTGTGCAAATAAATTTCAGGGTCAGTTTACCATAGGTGGCATCGCCCT | 55 |
| 5 | F | ATTTGCACAACCGGCAAACTGCCGGTTCCGTGGCCTACACTGGTTACCAC | 50 |
| 6 | R | CGGATAACGGCTAAAGCACTGAACACCATRTGTCAGGGTGGTAACCAGTGTAGG | 54 |
| 7 | F | GCTTTAGCCGTTATCCGGATCATATGAAACAGCATGATTTCTTCAAATCTGCAATGCCG | 59 |
| 8 | R | CGTCTTTGAAGAAAATGGTACGTTCCTGAACATAACCTTCCGGCATTGCAGATTTGAAGA | 60 |
| 9 | F | GTACCATTTTCTTCAAAGACGATGGTAATTATAAGACCCGCGCAGAAGTTAAAT | 54 |
| 10 | R | ACCTTTCAGCTCAATACGATTCACCAGGGTATCACCTTCAAATTTAACTTCTGCGCGG | 58 |
| 11 | F | CGTATTGAGCTGAAAGGTATTGATTTTAAAGAAGATGGCAATATTTTGGGTCATAAA | 57 |
| 12 | R | ATGTACACATTGTGGCTGTTAWAATTATATTCCAGTTTATGACCCAAAATATTGCC | 56 |
| 13 | F | CAGCCACAATGTGTACATTATGGCAGATAAACAAAAGAACGGTATTAAAGTGAATTTT | 58 |
| 14 | R | CTGCCATCCTCAATGTTGTGACGAATTTTAAAATTCACTTTAATACCGTTCTTTT | 55 |
| 15 | F | CAACATTGAGGATGGCAGCGTTCAGCTGGCGGACCATTATCAACAGA | 47 |
| 16 | R | GGTTATCAGGCAGTAAGACCGGGCCATCACCAATCGGGGTATTCTGTTGATAATGGTCCG | 60 |
| 17 | F | GTCTTACTGCCTGATAACCATTATCTGAGCACCCAGAGCGCACTGAGCAAGG | 52 |
| 18 | R | TCACAAATTCCAGCAGCACCATATGATCACGTTTCTCATTAGGATCCTTGCTCAGTGCGC | 60 |
| 19 | F | TGCTGCTGGAATTTGTGACCGCAGCAGGTATTACCCTGGGTATGGACGAACTGTACAA | 58 |
| 20 | R | GTTAGCAGCCGGATCCTTATTTGTACAGTTCGTCCATAC | 39 |

**F/R**: Forward/Reverse
